# Ultrasensitive Electrochemical Immunosensors Using Nanobodies as Biocompatible Sniffer Tools of Agricultural Contaminants and Human Disease Biomarkers

**DOI:** 10.3390/mi14081486

**Published:** 2023-07-25

**Authors:** Rodica Elena Ionescu

**Affiliations:** Light, Nanomaterials and Nanotechnology (L2n) Laboratory, CNRS EMR 7004, University of Technology of Troyes, 12 Rue Marie Curie CS 42060, 10004 Troyes, France; elena_rodica.ionescu@utt.fr; Tel.: +33-3-2575-9728; Fax: +33-3-2571-8456

**Keywords:** nanobodies, conductive supports, biofunctionalization strategies, electrochemical transductions, environmental toxicity, human biomarkers

## Abstract

Nanobodies (Nbs) are known as camelid single-domain fragments or variable heavy chain antibodies (VHH) that in vitro recognize the antigens (Ag) similar to full-size antibodies (Abs) and in vivo allow immunoreactions with biomolecule cavities inaccessible to conventional Abs. Currently, Nbs are widely used for clinical treatments due to their remarkably improved performance, ease of production, thermal robustness, superior physical and chemical properties. Interestingly, Nbs are also very promising bioreceptors for future rapid and portable immunoassays, compared to those using unstable full-size antibodies. For all these reasons, Nbs are excellent candidates in ecological risk assessments and advanced medicine, enabling the development of ultrasensitive biosensing platforms. In this review, immobilization strategies of Nbs on conductive supports for enhanced electrochemical immune detection of food contaminants (Fcont) and human biomarkers (Hbio) are discussed. In the case of Fcont, the direct competitive immunoassay detection using coating antigen solid surface is the most commonly used approach for efficient Nbs capture which was characterized with cyclic voltammetry (CV) and differential pulse voltammetry (DPV) when the signal decays for increasing concentrations of free antigen prepared in aqueous solutions. In contrast, for the Hbio investigations on thiolated gold electrodes, increases in amperometric and electrochemical impedance spectroscopy (EIS) signals were recorded, with increases in the antigen concentrations prepared in PBS or spiked real human samples.

## 1. Introduction

Nanobodies or VHH are found in sera of camelids (camels, llamas, and alpaca) [1,2], and are intensively used in diagnostics due to their improved stability in in vitro and in vivo conditions [3], non-invasive properties, imaging tissue characterization [4,5,6,7], and high penetration properties into solid tumors [8,9]). Nbs are successfully recommended for clinical treatments [10,11] and therapeutics [12], having a similar antigen (Ag) binding capacity to conventional Abs [13] but with higher affinity [14] for inaccessible epitopes [15]. Moreover, Nbs are prescribed in monitoring the evolution of several human diseases such as different types of inflammation, skin eczema, and blood/organ disorders within the detection of PSA (soluble Ag)/PSMA (membrane Ag) in blood/prostate tissue [16], viral [17,18,19,20] and non-viral infections [21,22], and allergy [23] symptoms.

**Figure 1:**
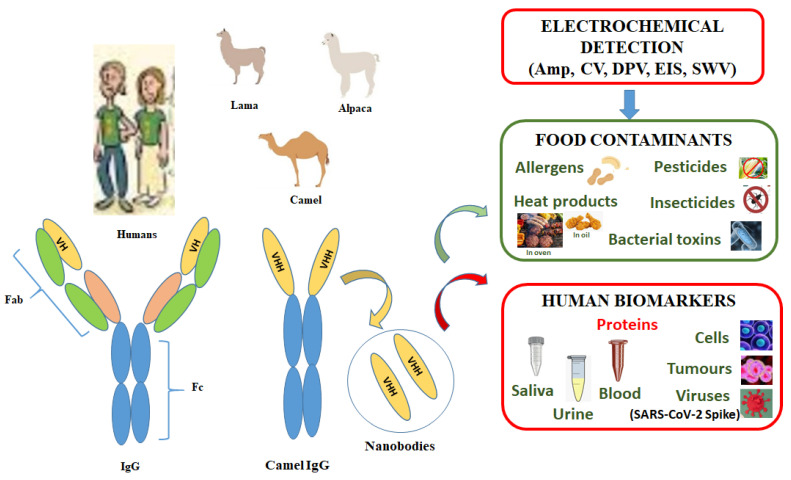
Electrochemical methods for the detection of food contaminants and human disease bio-markers. (Amp—amperometry, CV—cyclic voltammetry, DPV—differential pulse voltammetry, EIS—electrochemical impedance spectroscopy, SWV—square wave voltammetry; VHH—camelid family variable-single domain antibodies named nanobodies.

Nbs (~15 kDa) smaller that Abs (~150 kDa) are obtained by cloning the variable domain heavy-chain antibodies of camelids that make them attractive reagents for in vitro diagnostics [24], food toxins [25], environmental pollutants [26], optical immunoassays, and several types of biosensing platforms [27]. Unfortunately, Nbs are not available for a wide variety of target analytes [28] as they have a high uptake in the kidneys, causing nephrotoxicity [29]. For these reasons, in vitro detection of environmental contaminants and human biomarkers is preferred as much as possible (Figure 1).

Several electrochemical biosensors have been proposed for the detection of different classes of raw (e.g., Ara 1 allergens [30], triazophoros insecticides [31], ricin bacterial toxin [32]), and cooked (acrylamide [33]) food contaminants. However, problems have arisen regarding the robustness of biofunctionalization due to the inevitable temperature fluctuations that affect the activity of the whole Abs and/or enzyme label or prepared oligonucleotides sequences. Therefore, the biosensor stability, target limit of detection (LOD), and sensor selectivity in complex medium counter some difficulties. Similar problems are also noted for the electrochemical detection of human biomarkers in complex and/or spiked real samples using benchtop immunosensors for traces of SARS-CoV-2 spike protein [34], alpha fetoprotein (AFP) [35], and prostate specific antigen (PSA) [36]. To overcome these problems, Nbs with high thermal stability in the range of 50 °C to 90 °C offer an elegant and inexpensive solution for future generations of miniaturized biosensors. In addition, more devices connected to smartphones will continue to progress and play a major role in real-time monitoring of human health regardless of the region of the globe where temperatures can easily rise to 50 °C.

Currently, Fcont are often detected using immunosensor-based Nbs/proteins labeled with HRP enzyme related to target concentrations in the presence of active redox substrates in an aqueous electrolyte. In contrast, Hbio were detected with label-free immunosensors limiting the non-specific contamination of the electrode surface, avoiding possible problems with the activity/stability of HRP in complex fluids and temperature fluctuations.

In this review, the latest developments of electrochemical biosensors using Nbs immobilized on conductive supports for the detection of traces of various chemical and biological targets presented in body fluids (blood, saliva, and urine) and spiked aqueous solutions are herein discussed. Interestingly, Nbs are widely used in drug treatments, but not in the construction of electrochemical immunosensors as bioreceptors on solid surfaces.

## 2. Generation of Nanobodies

The smallest antibodies (Nbs of about 15 kDa) are composed of only heavy protein chains that provide high affinity and specificity for antigenic molecules. Nbs are generated through three types of libraries, named the immune (10^6^–10^8^ CFU/mL), the naïve (10^9^–10^11^ CFU/mL), and the synthetic library (10^9^–10^15^ CFU/mL) [37]. Thus, Nbs are obtained from immunized camelids with non-toxic antigens, while other alternative routes are considered for antigens that are highly toxic, pathogenic, or nonimmunogenic. In such situations, synthetic phage display library is the most common procedure to obtain Nbs with high specificity and affinity against harmful antigens. For example, 1.65 × 10^9^ CFU/mL is considered a large library for obtaining high affinity clones [38]. Fortunately, unique and robust Nbs can be efficiently expressed in large quantities at low cost, most likely in *Esche-richia coli* bacteria, and not in mammalian cells as required for full-size Abs, which is a much more complicated, temperature-dependent, and expensive approach. Nowadays, there are only a few examples of the use of Nbs for rapid electrochemical investigations of toxic compounds found in food products and bodily fluids reported in recent literature.

## 3. Immobilization Strategies of Nanobodies

Nbs have been covalently attached to conductive surfaces using two generic approaches referred to herein as direct and indirect tube/drop functionalizations based on classical enzyme-linked immunosorbent assay (ELISA) developed with whole Abs. To obtain a low limit of detection, the electrodes were often modified with biocompatible polymeric films decorated with different types of nanomaterials (AuNPs, NiONPs, SWCNTs/MWCNTs, and nylon fibers) which increased the density of Nbs on the surface of the working electrodes. Moreover, some works adopted the silane [39] and thiol chemistries [40] with two functionalization steps (Nbs/target Ags) that also provided performant immune-nanobody sensing schemes. Finally, an important parameter that validates the biofunctionalization steps on different types of electrodes concerned the choice of the redox probe and its optimal concentration, which was either freely present in an electrolyte solution, mixed with Nbs, or deposited on electrogenerated or colloidal nanomaterial-coated electrodes.

### 3.1. Direct Surface Functionalization

Substrates modified with gold layers or AuNPs are used for direct functionalization with Nbs carrying cysteine at the C-terminal end for 24 h [41], while HaloTagged-Nbs/proteins are often proposed for one-step biocoating of supports [42]. Interestingly, the SpyTag/SpyCatcher technology provides control over Abs/Nbs orientations [43] on gold electrodes modified with chemical self-assembled monolayers (SAM) made of synthetic SpyTag peptide on HDT monolayer [44]. In 2014, carbon-based support as SPCE was used in the development of the first sandwich nanoimmunoassay with linked capture Nbs though EDC/NHS chemistry for the detection of target human epidermal growth factor receptor (HER2) using a second Nbs-labeled horseradish peroxidase (HRP) [45,46]. Moreover, Nbs were successfully modified with lysine (Lys)/histidine (His) amino acids for selective one-step immobilization on supports [47].

### 3.2. Indirect “In Tube” and “In Drop” Surface Functionalization

Polythiophene modification of SPCE was carried out for tracking the immunocoplexes made of cancer biomarker epidermal growth factor receptor (EGFR) antigen and Nbs labeled-Fe_3_O_4_/*N*-trimethyl chitosan/AuNPs formed initially “in Eppendorf tube” [48]. Additionally, haptens coupled to protein carriers such ovalbumin (OVA) and bovine serum albumin (BSA) [49] were used for conjugation with Nbs, using “in drop” competition for binding to Nbs-HRP between free target and target haptens-OVA/BSA attached on SPCE supports. Moreover, a sensitive sandwich immunoassay using biotinylated Nbs was developed and applied to biotin-streptavidin-based ELISA [50] and electrochemical sensors.

## 4. Nanobodies for the Electrochemical Detection of Food Contaminants

**Figure 2:**
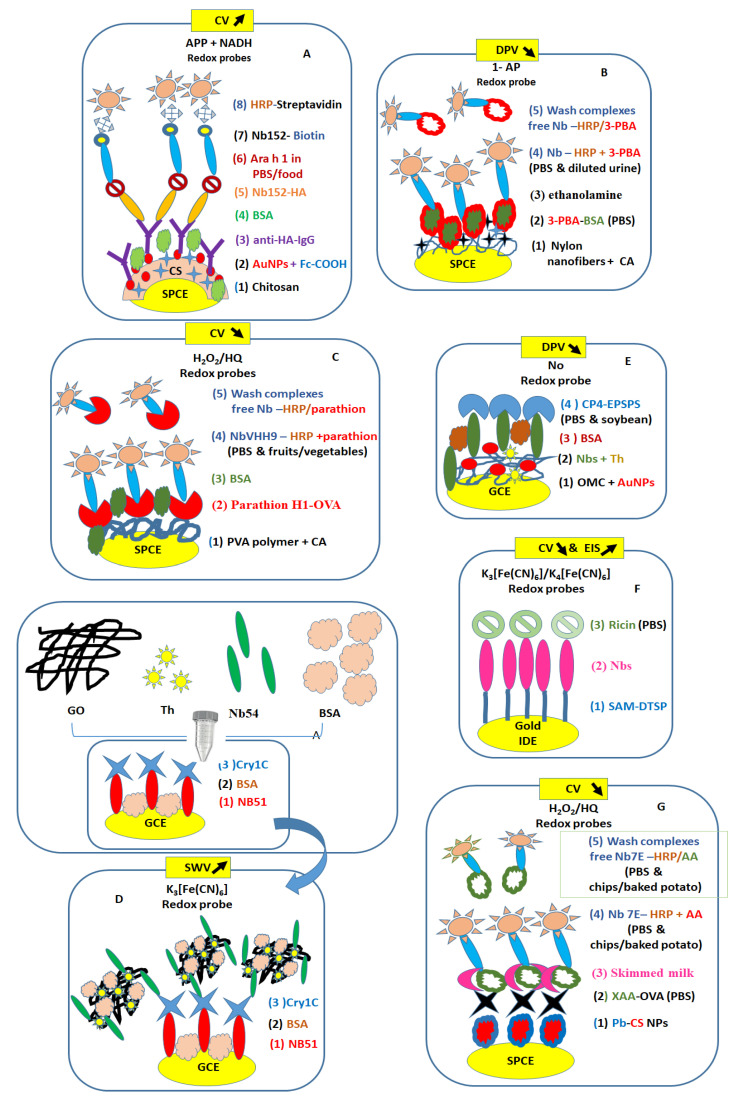
Electrochemical methods for the detection of food contaminants in PBS buffer and spiked real samples such as EIS (**F**), CV (**A**,**C**,**F**,**G**), DPV (**B**,**E**), and SWV (**D**). CA—citric acid; CP4-EPSPS—*Agrobacterium* sp. strain CP4 protein; CS—chitosan; GO—graphene oxide; OMC—ordered mesoporous carbon; DTSP—dithiobis (succinimidyl propionate); PVA—polyvinyl alcohol polymer; Pb—Prussian blue; SAM—self-assembled monolayer. (**A**) [52], (**B**) [54], (**C**) [55], (**D**) [57], (**E**) [58], (**F**) [60], and (**G**) [61]. For each electrochemical method, arrows indicate the upward or downward trend of the recorded signals for increasing concentrations of targets.

Recent research papers have mentioned the great potential in the use of camelid VHHs for monitoring environmental chemicals for safe agricultural production. In this section, some examples of toxic chemicals detected using optimal selection of Nbs and different electrochemical approaches on screen-printed carbon electrodes (SPCE) and gold-coated supports for efficient and effective biofunctionalization protocols are provided (Figure 2, Table 1).

### 4.1. Allergens

Peanuts may cause fatal allergic disease in humans. The responsible allergen molecules belong to different protein families named Ara 1–17, where Ara h 1–3 induce the highest allergic reaction to IgE. To monitor traces of Ara h 1 allergen, optimized Nbs-pair (Nb152-hemagglutinin-HA/Nb152 biotin-B) are proposed in the construction of an electrochemical CV immunosensor using SPCE modified with electrogenerated chitosan/gold nanoparticles (AuNPs) mixed with carboxyl-ferrocene/anti-HA IgG/BSA for staining with alkaline phosphatase conjugated streptavidin (SA-ALP). In the presence of usual p-aminophenol phosphate (APP) and NADH substrates for ALP enzyme [51], it was found that such a sensing scheme provided about 11 times higher sensitivity and shorter operation time than classical ELISA. Spiked milk and chocolate were confirmed with the above Nbs-based sensor [52].

### 4.2. Insecticides

Triazophos insecticides used in agricultural products in provenance from Asian and African countries were detected with one-step ELISA immunoassay. The authors used VHH T1 Nbs genetically fused with alkaline phosphatase (AP) that had a half-maximum inhibition concentration of 6.6 ng/mL triazophos. Authors mentioned negligible influence of cross-reactivity in the presence of organophosphate pesticides (<0.1%) and good average recoveries of triazophos from water, soil, and apple samples [53]. Interestingly, after contact with phrethroid insecticide, the toxic phenoxybenzoic acid (3-PBA) metabolite is formed in human urine. One study proposed the detection of 3-PBA in aqueous solutions (PBS and human urine) using differential pulse voltammetry (DPV) for an immunosensor scheme on SPCE. Specifically, 3-PBA-conjugated with BSA were covalently immobilized on SPCE modified with nylon nanofibrous membranes/citric acid (CA)/BSA-3-PBA and exposed to Nbs-ALP that compete with unbound 3-PBA in aqueous solutions (PBS/urine) in the presence of specific ALP substrate (1-naphthyl phosphate -1-NP). This immunosensor was tested in ten-fold diluted urine (0.01 to 0.5 ng mL^−1^ 3-PBA) [54].

### 4.3. Pesticides

Even though safety rules are ongoing, sometimes vegetables and fruits are contaminated with parathion, a banned organophosphorus pesticide. Therefore, there is urgent need of a sensitive biosensor method to detect parathion traces in food products (e.g., cabbages, cucumbers, and oranges). Herein, SPCE were laminated with polyvinyl alcohol polymer (PVA)/citric acid (CA) nanofibers. The resulting electrodes were used for covalent immobilization of parathion hapten (H1) conjugated with ovalbumin (OVA) for testing its affinity capability to horseradish peroxidase Nbs (VHH9-HRP), which compete with free parathion recorded by cyclic voltammetry (CV) showing changes of reduction peak current before and after the addition of H_2_O_2_ substrate. The immunosensor was tested in spiked food samples for parathion at 0.05 and 0.10 ng/g food [55].

### 4.4. Bacterial Toxins

Traces of toxin Cry1C protein produced by *Bacillus thuringiensis* bacteria [56] in genetically modified (GM) crops were detected on glassy carbon electrode (GCE) using a sandwich immunosensor with Nb51 for coating electrode and Nb54/graphene oxide (GO)-thionine (Th) for in-tube functionalization steps. In this study, different concentrations of Cry1C were prepared in PBS, while spiked corn extracts (0.1, 1, 10, 100 ng·mL^–1^) were successful detected using the SWV immunosensors. Additionally, the Cry1C immunosensor was operational in the pg/mL range compared to the ELISA-based mAb in the ng/mL range [57].

The 5-enolpyruvylshikimate-3-phosphate synthase (EPSPS) enzyme derived from *Agrobacterium* sp. strain CP4 protein (CP4-EPSPS) was introduced by genetic manipulation in GM crops with strong resistance to herbicide glyphosate. CP4-EPSPS protein was detected in PBS and spiked nontransgenic soybean samples (1, 10, 100 ng/mL) using electrochemical immunosensors constructed on GCE modified with ordered mesoporous carbon (OMC) decorated with AuNPs and Nbs mixed with Th redox probe, which monitored the current changes by differential pulse voltammetry (DPV). The sensitivity of such an immunosensor was at least three orders of magnitude higher than other methods and the commercial CP4-EPSPS kit [58].

Another toxin, ricin chain A, is produced in the castor oil plant, *Ricinus communis* [59], and was detected on dithiobis succinimidyl propionate (DTSP), formed a self-assembled monolayer (SAM) on gold coated interdigitated microelectrode (IDE), and functionalized with Nbs. The immunosensor proved advantages in terms of thermal stability and shelf-life of the Nbs over the conventional polyclonal and monoclonal antibodies when two hours for SAM formation was employed for maximum CV current peak responses in PBS (pH 7.4 with redox probes). EIS method was also employed for the detection of a single ricin concentration (1 pg/mL PBS). No data using real samples were reported [60].

### 4.5. Thermal Processing Product—Acrylamide

Acrylamide (AA) toxic product formed during heat treatment of foods was detected in PBS and spiked samples (potato chips and biscuits) using an electrochemical immunoassay monitoring the changes in electrocatalytic cathodic current of biofunctionalized SPCEs before and after the addition of H_2_O_2_ substrate. Thus, the electrodes were systematically modified with an electrogenerated film of Prussian blue chitosan nanoparticle (PB-CS–NP) film/xanthyl-derivatized acrylamide (XAA)-OVA/skimmed milk/diluted Nb-7E mixed with AA concentrations (1:40)/HRP-goat anti-VHH IgG. Such a sensing scheme has shown three-fold improvements in LOD over classical ELISA [61].

**Table 1:** Electrochemical methods using nanobodies as sniffer bioreceptors of food contaminants in saline buffer and spiked real samples.

Conductive Supports	Method	Redox Probe	Analyte	Nanobodies (Nbs)	Linear Range	Sensor Signal Stability	LOD	Ref
SPCE/ Chitosan/ AuNPs+ Fc-COOH	CV	0.1 mM APP, 2 mM NADH	Ara h 1 1,3,5,10, 15,30,50,100 ng/mL	Nb152-HA (100 μg/mL)/ Nb152-B (100 μg/mL)	4.5–55 ng/mL	15 days at 4 °C	0.86 ng/mL	[52]
SPCE/ nylon + CA	DPV	1 mg/mL 1-NP	3-PBA 8 × 10^−4^; 1 × 10^−3^, 5 × 10^−3^, 1 × 10^−2^, 5 × 10^−2^, 1 × 10^−1^, 5 × 10^−1^, 1 ng/mL & 3-PBA-BSA_CAT_ (10 μg/mL)	Nb-ALP (400 µg/mL)	0.8–1000 pg/mL	5 weeks at 4 °C	0.64 pg/mL	[54]
SPCE	CV (cathodic peak)	0.5 mM HQ/ 1.5 mM H_2_O_2_	Parathion 10^−2^–10^2^ ng/mL & parathion H_1_-OVA_CAT_ (5 µg/mL)	VHH9-HRP (9 µg/mL)	10^−2^–10^2^ ng/mL (8 conc.)	63 days for selected 0.1 and 1 ng/mL parathion after regeneration cycles	2.26 pg/mL	[55]
GCE	SWV	2 mM K_3_[Fe(CN)_6_]	Cry1C -protein (1, 10, 10^2^, 10^−1^, 10^−2^, 10^−3^ ng/mL)	Nb51 (100 µg/mL on electrode) & Nb54 (1 mg/mL added in GO-Th supernatant)	1, 10, 10^2^, 10^−1^, 10^−2^, 10^−3^ ng/mL	Nbs stable at 70 °C & Immuno-sensor stable for 15 days at 4 °C	3.2 pg/mL	[57]
GCE/OMC + AuNPs	DPV	N_2_-saturated 100 mM PBS pH 7.4	EPSPS - enzyme (1, 10, 10^2^, 10^−1^, 10^−2^, 10^−3^ ng/mL)	Nb (10 µg/mL) & Th (100 µg/mL)	1, 10, 10^2^, 10^−1^, 10^−2^, 10^−3^ ng/mL	Nbs stable at 70 °C (~60% activity) & Immunosensor stable over 14 days at 4 °C (~80% from initial activity at 10 ng/mL Ag)	0.72 pg/mL	[58]
Au-IDE	CV & EIS	5 mM K_3_[Fe(CN)_6_]/ K_4_[Fe(CN)_6_]	Ricin chain-A 10^−3^, 1, 10^3^, 10^6^ pg/mL & 1 pg/mL	Ricin Nbs (5.2 mg/mL)	10^−3^, 1, 10^3^, 10^6^ pg/mL	up to 40 °C with a shelf-life of 1 week	1 fg/mL	[60]
SPCE/chitosan NPs	CV (cathodic peak)	1 mM HQ/ 6% H_2_O_2_	AA (0.39, 0.78, 1.56, 3.125, 12, 5, 25, 50 µg/mL) & XAA-OVA_CAT_ (10 µg/mL)	Nb-7E (7.45 µg/mL, used at 1:40 dilution ~ 18 µg/mL)	0.39 to 50.0 μg/mL	bind to Ag at 95 °C due to four Cys involve in two disulfide bonds	0.033 μg/mL	[61]

*Abbreviations*: AA—acrylamide; APP—p-aminophenyl phosphate; ALP—alkaline phosphatase; BSA—bovine serum albumin; CA—citric acid; Fc-COOH—carboxyl ferrocene; CAT—electrode coating antigen; Cys—cysteine; EPSPS—5-enolpyruvylshikimate-3-phosphate synthase; HQ—hydroquinone; HRP—horseradish peroxidase; GO-Th—graphene oxide thionine; 3-PBA—3 phenoxybenzoic acid; H1-OVA—hapten 1 ovalbumin; OMC—ordered mesoporous carbon; 1-NP—1-naphthyl phosphate; 3-PBA—3-phenoxybenzoic acid; XAA—9-xanthyldrol-derivatized acrylamide

In conclusion, Nbs-based immunosensors for the detection of environmental contaminants showed very good stability over at least 15 days up to 63 days (Table 1), while it was noticed that, in the case of the thermoformed acrylamide product, the immune bin-ding events between Nbs-AgAA occurred at an abnormally high temperature of 90 °C, which is not possible with full-size Abs.

## 5. Nanobodies for the Electrochemical Detection of Human Biomarkers

**Figure 3:**
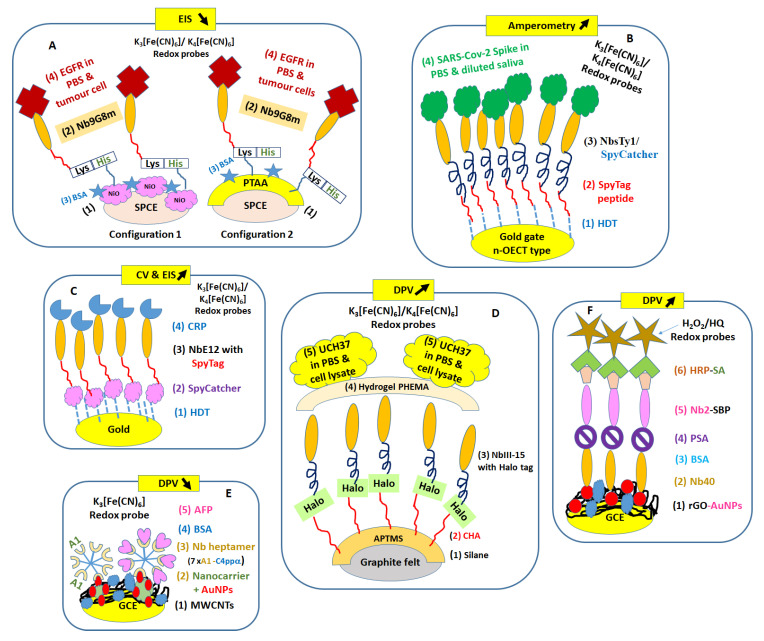
Electrochemical methods for the detection of human biomarkers in PBS buffer and spiked real samples such as EIS (**A**,**C**), CV (**A**), amperometry (**B**), and DPV (**D**–**F**). APTMS—3-aminopropyl)trimethoxysilane; CHA—6-chlorohexanoic acid, EGFR—epithelial growth factor receptor; HDT—1,6-hexanedithiol; HEMA—2-hydroxyethyl methacrylate; PHEMA—poly(2-hydroxyethyl methacrylate; PTAA—poly (thiophene acetic acid). (**A**) [62], (**B**) [64], (**C**) [67], (**D**) [70], (**E**) [72], (**F**) [76]. For each electrochemical method, arrows indicate the upward or downward trend of the recorded signals for increasing concentrations of targets.

In recent years, few works have been reported on the electrochemical detection of human biomarkers using nanobodies. In this section, bioreceptors of tumors such as glycoproteins and various proteins present in complex body fluidics are detected using different types of Nbs and electrochemical methods. As expected, the SPCE electrodes and gold-coated supports were also used for performant biofunctionalization protocols (Figure 3, Table 2).

### 5.1. Glycoproteins in Tumour Cells

The transmembrane glycoprotein located at the cell surface named epithelial growth factor receptor-EGFR is presented in different tumor cell lines such as SW480 (stage II) and SW620 cell lines derived from a colon adenocarcinoma and a lymph node metastasis. This protein was detected with a label-free EIS immunosensor on SPCE support modified with either electrodeposited nanostructures (NiO NPs) or with electrogenerated poly (thiophene acetic acid) film- PTAA) in about 60 min. Such supports were used in immobilized orientation-controlled nanobodies due to their modification with short peptide tail with lysine (Lys)/histidine (His) named Nb9G8m. In this study, EIS monitored the resistance to electron transfer of different EGFR concentrations in saline buffer (PBS at pH 7.4 containing the redox probe) providing low LODs for two immunosensor configurations. Moreover, EGFR in the whole membrane of three tumor cell lines (1 × 10^6^ each cell line/mL) was detected with both sensing platforms, confirming that A431 cells collected from epidermoid carcinoma expressed the highest level of EGFR, followed by SW480 cells (from colon adenocarcinoma), SW620 cells (from a lymph node metastasis), and HEK293 cells (non-neoplastic control cell line) [62].

### 5.2. Proteins in Complex Media—Saliva, Cell Lysate, Serum, Urine

Saliva is a preferred medium for diagnostic applications due to its simplicity/non-invasive collection [63] which minimizes the interaction between patients and medical staff. An alternating current electrothermal flow (ACET)-integrated n-type organic electrochemical transistor (OECT)-based immunosensor was proposed for 2 min detection of SARS-CoV-2 spike proteins in PBS and 4× diluted saliva. The amperometric immunosensor used a gate electrode with 100 nm Au coating on glass substrate which was systematically (bio)functionalized with 1,6-hexanedithiol (HDT)/maleimide-modified SpyTag peptide solution in PBS (0.1 mg/mL)/SpyCatcher Ty1-Nbs (for specific S protein bindings) or GFP- Nbs (for control)/S-target. In this work, the immunoreactions occurred at RT over 30 min before electrochemical investigations using 100 nW power consumption for n-type OECT-sensing platform with 100 nA drain current and 100 mV saturation voltage regime [64].

The SpyTag/SpyCatcher protein conjugation system was also used for the electrochemical detection of C-reactive protein (CRP), a sensitive serum biomarker of inflammatory/infectious processes, including cardiovascular diseases [65]. In healthy individuals, the concentration of CRP should be below 5 µg/mL [66]. In this work, CRP prepared in PBS buffer was tested with CV/EIS methods using the affinity of recombinant nanobodies NbE12 fused to SpyTag immobilized on SpyCatcher coated gold electrode. So far, all immunodetections concerned only five CRP concentrations. No data with real samples were provided [67].

Nanobody NbIII.15, derived from a synthetic yeast surface display library, was engineered to bind with high affinity and specificity to the human enzyme UCH37—a predictor biomarker of the hepatocellular carcinoma [68] in heterogeneous media, such as cell lysate, without added purification or preconcentration steps. For DPV investigations, graphite felt was modified with 3-aminopropyl)trimethoxysilane (APTMS)/6-chlorohexanoic acid (CHA) and freshly used for covalent binding of recombinant NbIII.15 modified with versatile HaloTag protein labelling [69], and further encapsulated into a photogenerated thin hydrogel layer made of poly (2-hydroxyethyl methacrylate) (PHEMA) that created an homogenous coating matrix on the graphite sheet. The use of such a polymer was essential to minimize false positive signals due to nonspecific biomolecules adsorption from complex composition of saliva. This immunosensor was tested using DPV method, which displayed current decreases upon antigen binding to NbIII.15 in bench prepared concentrations in PBS and into unpurified HEK293 cell lysate without the need to include the redox probe [70].

Alpha fetoprotein (AFP) is the most important used and accepted serum predictive biomarker of hepatocellular carcinoma (HCC), with 20 ng/mL for HCC screening and diagnosis or 200 and/or 400 ng/mL for treatment stratification [71]. Recently, traces of AFP in the pg/mL range were detected with an electrochemical immunosensor based on Nbs A1-C4bp α heptamer with 90 min incubation time on GCE modified with AuNPs on ZIF-8 as an interesting nanocarrier that enhanced the heptamer loading at the electrode surface. This immunosensor showed good selectivity in the presence of prostate-specific antigen (PSA), carcinoembryonic antigen (CEA), and neuron-specific enolase (NSE) interfering proteins (each of them at 10 ng/mL) when AFP showed DPV peak current change of about 220 μA versus 30 μA for the other three proteins [72].

Detection of prostate cancer implies the presence of prostate specific antigen (PSA) in serum samples [73] and of annexin A3 (ANXA3) protein (<1 fg/mL) [74] as a noninvasive urine biomarker [75]. Several sensing methods were proposed using conventional anti-PSA antibodies but very few used ANXA3 and even fewer used Nbs. For example, different levels of PSA in the nanomolar range were detected with an electrochemical immunosensor using a sandwich pair of Nb40/Nb2 with fused SBP tag nanobodies on GCE initially modified with reduced graphene oxide (rGO)/AuNPs. The proposed immunosensor proved good selectivity in the presence of interfering compounds such as HSA, human IgG, vitamin C, and glucose, and good correlation within positive real serum samples [76].

**Table 2:** Electrochemical methods using nanobodies as sniffer bioreceptors of human biomarkers in saline buffer and spiked real samples.

Conductive Supports	Method	Redox Probe	Analyte (Ag)	Nanobodies (Nbs)	Linear Range	Sensor Signal Stability	LOD	Ref
SPCE/ NiO NPs & SPCE/ PTAA film	EIS	5 mM K_3_[Fe(CN)_6_]/ K_4_[Fe(CN)_6_]	EGFR 0.25, 1, 5, 10, 15, 25, 50 μg/mL^−1^ (on NiO NPs) & EGFR 0.5, 1, 5, 10, 15, 25, 50 μg/mL^−1^ (on PTAA)	Nb9G8m with Lys/His (50 μg/mL)	0.25 to 50 μg/mL (on NiO NPs) & 0.5 to 50 μg/mL(on PTAA)	up to 40 °C with shelf-life of 1 week	0.48 µg/mL (NiO NPs) & 1.14 µg/mL (PTAA)	[62]
Glass/Au/ HDT	Amperometric	10 mM K_3_[Fe(CN)_6_]/ K_4_[Fe(CN)_6_]	SARS-CoV-2 spike 10^−9^, 10^−10^, 10^−11^, 10^−12^, 10^−13^ 10^−14^, 10^−15^, 10^−16^, 10^−17^, 10^−18^ M (in PBS) & SARS-CoV-2 spike 3 × 10^−7^, 3 × 10^−9^, 3 × 10^−12^, 3 × 10^−15^, 3 × 10^−17^ M (in 4 × dil. saliva)	on n-OECT gate sensor:Nbs Ty1with SpyCatcher linker 20 × 10^−6^ M on n-OECT gate sensor & Nbs GFP1 × 10^−9^ M (as control)	NA	Current stability over 1 h	10^−16^ M	[64]
Gold	CV & EIS	5 mM K_3_[Fe(CN)_6_]/ K_4_[Fe(CN)_6_]	CRP 0.25; 0.35; 0.5; 1; 1.50 µg/mL (for CV/EIS)	NbE12 with SpyTag linker 2.5 µg/mL	0.25; 0.35; 0.5; 1µg/mL (for CV/EIS)	NA	0.21 µg/mL	[67]
Graphite felt	DPV	5 mM K_3_[Fe(CN)_6_]/ K_4_[Fe(CN)_6_]	UCH37 3 × 10^1^, 1 × 10^2^, 2.5 × 10^2^, 5 × 10^2^, 1 × 10^3^, pmoL (in PBS) & ~ 50 pmol UCH37/mL PBS in 1 mg cell lysate	NbIII.15 with HaloTag protein tail (5 × 10^6^ M)	NA	NA	25–30 pmol in PBS & cell lysate	[70]
GCE/ nanocarrier	DPV	5 mM K_3_[Fe(CN)_6_]	AFP 1, 10, 10^2^, 10^−1^, 10^−2^, 10^−3^, 10^−4^ ng/mL(in PBS) & 6.289, 12.564, 37.320, 72.693 ng/mL (in 4 human sera 10× dil. with PBS)	NbA1-C4bp α with Cys tail assembled intoheptamer structure (10 μg/mL)	1, 10, 10^2^, 10^−1^, 10^−2^, 10^−3^, 10^−4^ ng/mL	14 days at 4 °C	0.033 pg/mL in PBS	[72]
GCE/rGO +AuNPs	DPV	1 mM HQ, 10 mM H_2_O_2_ in deaerated PBS	PSA 10^−1^, 5 × 10^−1^, 1, 2.5, 5, 7.5, 15, 2 × 10, 3 × 10, 5 × 10, 10^2^ ng/mL	Nb40 on sensor (80 µg/mL) & Nb2-SBP for sandwich (90 µg/mL)	10^−1^ to 10^2^ ng/mL	over 4 weeks at 4 °C	0.08 ng/mL	[76]

*Abbreviations*: AFP—alpha fetoprotein; A1—AFP nanobodies A1; C4bpα—C-terminal fragment of C4-binding protein; CRP—protein C reactive; Lys—lysine; HDT—1,6-hexanedithiol; His—hexahistidine tag (6xHis-tag); n-OECT—n-type organic polymer electrochemical transistor; NiO NPs—nickel oxide nanoparticles; PTAA—poly(thiophene acetic acid) (PTAA); GFP—green fluorescence protein; UCH37—ubiquitin C-terminal hydrolases, one sub-family of de-ubiquitinating enzyme, also called UCH-L5; MWCNTs—multiwalled carbon nanotube with lengths of 10∼30 μm; nanocarrier—AuNPs decorated zeolitic imidazolate matrix; rGO—reduced graphene oxide; SBP—streptavidin-binding peptide.

Interestingly, it was noticed that the concentrations of Nbs used in the construction of immunosensors are much lower than those of standard Abs, namely 2.5 μg/mL versus 90 μg/mL (Table 2). Moreover, the conjugation time is often fixed at 1 h (Nbs) at 25 °C versus 12 h (Abs) at 4 °C.

## 6. Conclusions and Perspectives

Due to their superior chemical and physical properties, single-domain nanobodies as biorecognition probes will continue to interest researchers in developing the next generation of biosensors with high benefits for commercialization and in situ lateral flow immunoassays (LFAs) compatible with those using full-size Abs conjugated to colloidal nanomaterials. These inexpensive and dual visual ultrasensitive electrochemical methods will focus on the detection of analytes in complex food products and body fluid matrices.

Currently, there are only a few electrochemical biosensing schemes using mono-/multimeric Nbs for the detection of contaminants in food products and human biomar-kers spiked in PBS-diluted samples (saliva, urine, and blood). This review provides readers with currently validated Nbs-based immunodetection schemes in PBS buffer and spiked real samples using amperometry, CV, DPV, EIS, and SWV methods. As expected, carbon and gold-based materials were used as supporting electrodes. Moreover, their surfaces in several studies were nanostructured to provide higher binding site density to Nbs on the support and therefore increase the electrochemical sensing performance in terms of high sensitivity/specificity, low limit of detection, rapid sensor response, wide linear range, high sensor stability, as well as shelf-life (at least more than 14 days).

Despite their significant advantages over full-size antibodies, the production of Nbs for clinical applications using electrosensors is somewhat limited, due to either the lack of access to animals that generate Nbs, missing skills to express them in bacteria, or their commercial unavailability. Therefore, well-established classical Abs production is still preferred by researchers and companies interested in medical applications where wide varieties of analytes are either poorly soluble in aqueous solutions or highly toxic for safe handling. In this regard, efforts to design miniaturized sensing platforms will balance the limitations of Nbs accessibility and bring future developments to large-scale electroche-mical sensing with smartphones.

In addition, Nbs and their ultra-performant and accessible epitopes will be strategically used in the development of vaccines that will efficiently prevent mortality from pandemics (e.g., SARS-CoV-2 virus [77,78]) and greatly contribute to successful treatments of various infections/brain diseases, cancers, and drug intracellular deliveries [79]. The impact of nano technologies [80,81] on conventional electrochemical methods combined with artificial intelligence [82], sandwich enzyme-linked immunosorbent assay-based synthetic Nbs, and combinatorial binders enabling selection for the detection of small (bio)molecules [83] will provide a cutting edge for rapid and efficient selective diagnostics [84,85] and therapeutics [86,87].

## Data Availability

Not applicable.
